# Graphical Abstracts

**DOI:** 10.2174/1570159X1203140511151656

**Published:** 2014-05

















